# Clinical outcomes and costs for people with complex psychosis; a naturalistic prospective cohort study of mental health rehabilitation service users in England

**DOI:** 10.1186/s12888-016-0797-6

**Published:** 2016-04-07

**Authors:** Helen Killaspy, Louise Marston, Nicholas Green, Isobel Harrison, Melanie Lean, Frank Holloway, Tom Craig, Gerard Leavey, Maurice Arbuthnott, Leonardo Koeser, Paul McCrone, Rumana Z. Omar, Michael King

**Affiliations:** Division of Psychiatry, University College London (UCL), Maple House, 149 Tottenham Court Road, London, W1T 7NF UK; Camden and Islington NHS Foundation Trust, London, NW1 OPE UK; Department of Primary Care and Population Health, UCL, Rowland Hill Street, London, NW3 2PF UK; UCL PRIMENT Clinical Trials Unit, Research Department of Primary Care & Population Health, Royal Free Campus, Rowland Hill Street, London, NW3 2PF UK; South London and Maudsley Hospital NHS Foundation Trust, Bethlem Royal Hospital, Monks Orchard Road, Beckenham, BR3 3BX UK; Health Services Research Department, Institute of Psychiatry, Psychology and Neuroscience, King’s College London, De Crespigny Park, London, SE5 8AF UK; Bamford Centre for Mental Health and Wellbeing, University of Ulster, Northland Road, Derry, BT48 7JL UK; North London Service User Research Forum, Division of Psychiatry, UCL, Maple House, 149 Tottenham Court Road, London, W1T 7NF UK; Centre for the Economics of Mental and Physical Health, Institute of Psychiatry, Psychology and Neuroscience, King’s College London, De Crespigny Park, Derry, SE5 8AF UK; UCL Department of Statistical Science, Gower Street, London, WC1E 6BT UK

## Abstract

**Background:**

Mental health rehabilitation services in England focus on people with complex psychosis. This group tend to have lengthy hospital admissions due to the severity of their problems and, despite representing only 10–20 % of all those with psychosis, they absorb 25–50 % of the total mental health budget. Few studies have investigated the effectiveness of these services and there is little evidence available to guide clinicians working in this area. As part of a programme of research into inpatient mental health rehabilitation services, we carried out a prospective study to investigate longitudinal outcomes and costs for patients of these services and the predictors of better outcome.

**Method:**

Inpatient mental health rehabilitation services across England that scored above average (median) on a standardised quality assessment tool used in a previous national survey were eligible for the study. Unit quality was reassessed and costs of care and patient characteristics rated using standardised tools at recruitment. Multivariable regression modelling was used to investigate the relationship between service quality, patient characteristics and the following clinical outcomes at 12 month follow-up: social function; length of admission in the rehabiliation unit; successful community discharge (without readmission or community placement breakdown) and costs of care.

**Results:**

Across England, 50 units participated and 329 patients were followed over 12 months (94 % of those recruited). Service quality was not associated with patients’ social function or length of admission (median 16 months) at 12 months but most patients were successfully discharged (56 %) or ready for discharge (14 %), with associated reductions in the costs of care. Factors associated with successful discharge were the recovery orientation of the service (OR 1.04, 95 % CI 1.00–1.08), and patients’ activity (OR 1.03, 95 % CI 1.01–1.05) and social skills (OR 1.13, 95 % CI 1.04–1.24) at recruitment.

**Conclusion:**

Inpatient mental health rehabilitation services in England are able to successfully discharge over half their patients within 18 months, reducing the costs of care for this complex group. Provision of recovery orientated practice that promotes patients’ social skills and activities may further enhance the effectiveness of these services.

## Background

Inpatient mental health rehabilitation services in the UK provide specialist, tertiary care to people whose needs are so complex that they have not been able to be discharged from a standard inpatient mental health unit. Most have a diagnosis of schizophrenia or schizoaffective disorder with additional problems that compromise recovery. These include: inadequate response to usual antipsychotic medication, which occurs in up to 30 % [1]; cognitive impairment (executive function, verbal memory) and pervasive negative symptoms such as amotivation and blunted affect [2–4]; and co-existing issues such as substance misuse and challenging behaviours [5]. The complex nature of such problems generally lead to lengthy admissions and high support needs upon leaving hospital. Although they comprise only 10–20 % of those with psychosis, this group absorbs 25–50 % of the total mental health budget [6]. Thus, they are a low volume, high needs group. Despite this, there has been little research investigating the effectiveness of mental health rehabiliation services and the specific aspects of care that promote recovery and successful community discharge. Early studies of patient outcomes post deinstituitionalistaion suggested that the majority of people did well [7], and even those considered most “difficult to place” in the community remained well without requiring readmission at 5 year follow-up [8]. However, this group may not be representative of users of contemporary mental health rehabilitation services. This study comprised one part of a 5 year programme of research funded by the UK’s National Institute of Health Research into inpatient mental health rehabilitation sevices (the Rehabilitation Effectiveness for Activities for Life, or REAL study). The aim of this phase of the research programme was to investigate, prospectively, outcomes and costs for patients of better quality inpatient mental health rehabilitation services and to identify the components of care associated with clinical outcomes.

## Methods

The REAL study was approved by the South East Essex Research Ethics Committee (Ref. 09/H1102/45) and began in April 2009. The research was conducted in keeping with usual research governance guidance and local approvals were gained at each site. In the first phase, we contacted all NHS Mental Health Trusts in England to participate in a national survey of inpatient mental health rehabilitation services and a response rate of 87 % was achieved, involving 133 individual inpatient units [9]. These units were assessed using the Quality Indicator for Rehabilitative Care (QuIRC), a web based toolkit, completed by service managers (available at www.quirc.eu), which reports on seven domains of care in longer term units for people with complex mental health problems: Living Environment; Therapeutic Environment; Treatments and Interventions; Self-Management and Autonomy; Social Inclusion; Human Rights; Recovery Based Practice. The QuIRC has excellent inter-rater reliability [10] and good internal validity [11]. It can be completed in around 45 min and comprises 145 questions about: service provision (e.g. number of beds, average length of stay, built environment, treatments and interventions, staffing, staff turnover, training, supervision and disciplinaries); links with community organisations (e.g. colleges, employment agencies, sport and leisure facilities); the therapeutic milieu and recovery based practices (e.g. collaborative care planning, service user involvement, promotion of service users’ independent living skills); the protection of service users’ human rights (e.g. their privacy and dignity, their legal rights and the use of restraint and seclusion).

The 67 units that scored above the median on the QuIRC assessment in our national survey of inpatient mental health rehabiliation units in England [9] were considered eligible for this prospective study. We included units that scored above the median for quality in order to identify aspects of good quality care that might be associated with better patient outcomes. All those who were patients of these services during the recruitment phase of the study (July 2011 to December 2012) were eligible for inclusion, with the exception of those who were on leave (or had absconded) from the unit at the time of recruitment, those who lacked adequate English to give informed consent and those who were occupying a respite bed rather than a rehabiliation bed in the unit. Potential participants were approached by the researchers (IH, NG), after an initial introduction by a member of the unit’s clinical team, to explain the purpose and process of the study. A participant information sheet was provided and they were invited to ask questions about the study and given time to consider their involvement. Those who were assessed as having capacity to give informed consent but declined to participate were not recruited. However, the approval gained from the South East Essex Research Ethics Committee for the REAL study allowed inclusion of participants who lacked capacity to give informed consent to participate. Since mental health rehabilitation services focus on people with complex psychosis, this approval was important in preventing selection bias since we expected a proportion of patients to have significant cognitive impairment and ongoing positive symptoms of psychosis which would impar their capacity to consent to participate.

### Measurement of outcomes

Our primary outcomes assessed at 12 month follow-up were:

1. Social function as measured by the Life Skills Profile [12]
2. Length of admission in the rehabiliation unit
3. Successful community discharge i.e. without readmission or community placement breakdown. (Patients considered “ready for discharge” who were awaiting a vacancy in suitable accommodation were also included in a separate analysis to account for this potential confounder).

This study did not involve research interviews with patient participants. All data were gathered from case notes and interviews with unit staff. This approach was pragmatic given the fact that the two researchers (NG and IH) were concurrently recruiting and interviewing patients in a separate phase of the REAL study and it minimised the burden of research interviews on patients. Participant data were gathered at recruitment by the researchers from review of case notes as follows: demographics (age, gender, ethnic group); diagnosis; length of history; length of current admission; previous admissions; previous involuntary admissions; risk history. The researchers carried out face to face interviews with a member of the unit staff who knew each participant well (such as their primary nurse) to complete the following standardised measures: social function was assessed using the Life Skills Profile [12] which comprises 39 items, each rated on a four point likert scale with the most positive response scoring 4 and the least scoring 1, giving an overall score ranging from 39 to 156; engagement in activities was assessed using the staff rated version of the Time Use Diary [13] which assesses patients’ activities for the previous week during four periods each day - morning, lunchtime, afternoon and evening. The level of engagement in activity, and its complexity, is rated on a scale of 0 to 4 for each time period, giving a maximum possible score of 112 with higher scores denoting greater activity; patients’ function was assessed using the Global Assessment of Functioning [14] which provides an overall rating from 1 to 100 with higher scores denoting better functioning. Potential mediators of outcomes were also assessed using standardised measures that staff completed in face to face interviews with the researchers. Patients’ use of substances was assessed using the Clinician Alcohol and Drug Use Scales [15] and any challenging behaviours were assessed using the Special Problems Rating Scale [16]. Quality of care in the unit was rated by the unit manager using the QuIRC [10, 11] (staffing of the unit and availability of specific interventions are included in the QuIRC).

At 12 month follow-up, the researchers contacted a key informant to clarify whether the patient had been successfully discharged and if so, details of community placements were recorded. Where the patient had been discharged from the inpatient unit, the key informant was either the patient’s community care co-ordinator or their keyworker at their supported accommodation and for those who remained in the inpatient unit at follow-up, it was their primary nurse. Key informants were also asked to complete the Life Skills Profile [12] to re-assess social functioning. Data on length of admission in the rehabilitation unit were available from the unit manager and case notes and therefore available for all patients recruited to the study.

### Measurement of service use and costs

We used an adapted version of the Client Service Receipt Inventory [17] to collect information on the number of contacts with health care professionals in the unit and in the community over the last month at both time points (baseline and 12 month follow-up). We used data compiled by Curtis [18] to cost these service contacts assuming that each had an average duration of 30 min unless information from a comparable study was available to suggest otherwise [19].

### Data management

Data were entered into the study’s Access database by the researchers. Range and logic checks were built in to assist with data cleaning. Ten percent of data were double entered to check for data entry errors with an error rate set at 5 %, above which all data would be double entered. The error rate was less than 5 % and thus no double data entry was required. Data were further checked and cleaned by the statistician before analysis.

### Data analysis

Data were analysed using Stata version 12 [20]. Descriptive characteristics (demography and clinical and service characteristics) were summarised using mean (SD), median (interquartile ranges) or proportions as appropriate at baseline and 12 month follow-up. Summary statistics were calculated for all standardised assessments at baseline and follow up, providing percentages or mean (SD), median (interquartile ranges) as appropriate.

Random effects regression models were used to investigate the relationship between patient characteristics, service factors (QuIRC domain scores) and outcomes. This type of regression modelling takes account of clustering of patients within units [21]. Length of stay was log transformed due to violation of normality assumptions as assessed by residual plots. When fitting the models we followed the rule of 10 events per variable [22] to ensure that the coefficients were estimated with adequate precision. Results are reported as estimates with their 95 % confidence intervals. Complete case analysis was performed, meaning that only service user participant data and unit data were included in the analsyis where there were no missing values [23]. The results are treated as exploratory since no specific hypotheses were being tested.

A further intuitive analysis was carried out to identify service and patient factors that were associated with successful discharge and a composite outcome combining those successfully discharged with those considered by staff to be ready for discharge but awaiting a suitable community placement. Categorical or binary explanatory variables with less than 5 % prevalence were omitted from this analysis to avoid model fitting problems. Univariable analysis of prespecified factors considered to be potentially associated with the outcome of interest was first carried out. Variables with a *p*-value of less than or equal to 0.15 were considered in the mutivariable model. Backwards elimination was then carried out with a significance level of 5 %.

We compared baseline and follow-up cost of contacts and estimated the statistical significance of the change in total costs over time using a non-parametric bootstrap approach because of the skewed cost distribution. In addition, we estimated the association between baseline service user characteristics and baseline and follow-up cost of service contacts using a random effects regression model with robust standard errors, with latter used to also address the skewed data.

## Results

**Fig. 1:**
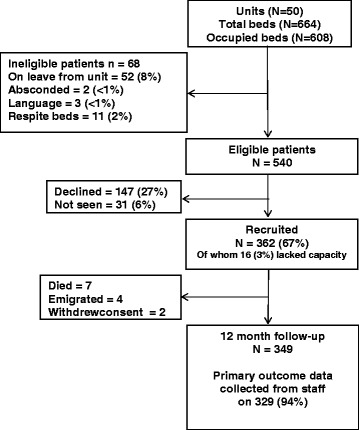
Participant recruitment and 12 month follow-up

Fifty of the 67 units (75 %) that scored above the median total QuIRC in our national survey of inpatient mental health rehabiliation units agreed to participate in the study. Within these units there were 540 potentially eligible participants, of whom 346 gave informed consent to participate and 16 who lacked capacity to do so were also recruited, giving an overall recruitment of 67 %. At 12 month follow-up, 7 participants had died, 4 had emigrated abroad and 2 had withdrawn consent. Of the remaining 349, the researchers gathered data on the three primary outcomes at 12 month follow-up on 329 (94 %). Figure 1 shows the participant flows in the study.

Two thirds of participants (65 %) were male, the majority (90 %) were white and most had a diagnosis of schizophrenia (68 %). Participants’ median length of contact with mental health services was 12 years and they had a median of four previous admissions, two of which had been involuntary. The median length of current admission was 18 months at recruitment, with seven of those months spent in the rehabilitation unit. Over two-thirds (71 %) had been detained involuntarily during the current admission and around half were still detained. One fifth (20 %) had previously been an inpatient in a secure unit and 9 % were currently detained on a “forensic” section of the Mental Health Act. The majority (81 %) had never been in prison but four (1 %) had been within the last 2 years. Just under half had physically assaulted other/s at some time; this had occurred within the last 2 years for 20 %. Serious assaults (resulting in the victim requiring hospital treatment or homicide) were uncommon (5 %) but four participants had killed somebody, all four incidents having occurred over 2 years ago. More prevalent was the risk of self-neglect and self-harm (65 % and 17 % respectively within the last 2 years). Mean staff ratings of service users’ functioning and activity were low, reflecting the severity of illness in this group. Problematic substance use was relatively uncommon (alcohol 7 %, illicit substances 4 %) (Table 1).

**Table 1:** Participant characteristics

	*N* = 362	
	n or mean	% or (SD)
Socio demographics and diagnosis		
Male	235	65
Age	39	(13)
White	324	90
Schizophrenia	238	68
Schizoaffective disorder	36	10
Bipolar disorder	26	7
Other	49	14
Contact with mental health services		
Time since first contact (years) median (IQR)	12	(6, 20)
Previous admissions median (IQR)	4	(2, 7)
Previous involuntary admissions median (IQR)	2	(0, 4)
Length of current admission (months) median (IQR)	18	(9, 38)
Length of current admission in rehabilitation unit (months) median (IQR)	7	(3, 15)
Current admission involuntary	250	71
Currently detained involuntary	174	49
Previous admission to special hospital (ever)	9	3
Previous admission medium secure unit (ever)	30	9
Previous admission low secure unit (ever)	70	20
Currently detained on forensic section	32	9
Of whom - Section 37	18	56
Of whom - Section 37/41	14	44
Risk history		
Assault on others more than 2 years ago	96	28
Assault on others within the last 2 years	68	20
Serious assault more than 2 years ago	20 (4 homicides)	5
Serious assault in last 2 years	4	1
Sexual offence more than 2 years ago	17	5
Sexual offence in the last 2 years	3	1
History of fire setting more than 2 years ago	15	4
History of fire setting in the last 2 years	10	3
Overdose or self-harm more than 2 years ago	83	24
Overdose or self-harm in the last 2 years	58	17
Recurrent self-harm in the last 2 years	21	6
Self-neglect in the last 2 years	220	65
Function		
Mean Global Assessment of Functioning rating	53	(8)
Mean Life Skills Profile rating	128	(15)
Mean Time Use Diary (activity) rating	49	(11)
Challenging behaviours		
Special Problems Rating Scale median (IQR)	0	(0, 2)
Problematic alcohol use	24	7
Problematic substance misuse	15	4

mean used when data Normally distributed, median when skewed

Table 2 shows the characteristics of the 50 units that participated in the study. Most were community based (88 %) and located in suburban areas (86 %) and the median bed number was 13. All units were staffed by nurses, support workers and psychiatrists and most had an occupational therapist and a psychologist on the team or access to these disciplines. The proportion of units with a psychologist increased over the period between participant recruitment and follow-up from 26 % to 36 %. Over one third of units (38 %) employed ex-patients on the staff team. With regard to the treatments and interventions offered, over one third of patients (37 %) were prescribed clozapine and none was prescribed more than two antipsychotics. Most (76 %) had an informal carer (a family member or friend) involved in their care, but family interventions had been carried out with only 7 % of service users in the previous 12 months. A total 13 % of patients had received CBT in the 12 months before recruitment. When unit managers were asked to rate the degree to which they felt comfortable with a close friend or relative of theirs receiving treatment in the unit, over half were “very happy”. The quality of the units, as assessed by the seven QuIRC domains, remained above the mean for all units in England throughout the 12 months [7].

**Table 2:** Unit characteristics

	Baseline		Follow up	
	n or median	% or (IQR)	n or median	% or (IQR)
Unit location				
Inner city	5	10		
Suburbs	43	86		
Rural area	2	4		
Unit type				
Hospital ward	6	12		
Community based	44	88		
Mean (SD) Mental Illness Needs Index score for local area of unit	1.09	(0.36)		
Staffing				
Psychiatrist works in the unit	47	94	50	100
Access to a psychiatrist	3	6	0	0
Clinical psychologist works on the unit	26	52	36	72
Access to a clinical psychologist	20	40	11	22
Occupational therapist works on the unit	39	78	41	82
Access to an occupational therapist	7	14	6	12
Nurse works on the unit	50	100	50	100
Support worker works on the unit	50	100	50	100
Social worker works on the unit	1	2	2	4
Access to a social worker	39	78	45	90
Ex-service user(s) work in the unit	19	38	20	40
Ex-service user(s) on the payroll	15	79	17	85
Staff turnover	14	(8, 21)	9	(4, 17)
Staff: service user ratio	1.8	(1.6, 2.2)	1.9	(1.8, 2.3)
Beds				
Beds on the unit	13	(10, 16)	13	(10, 16)
% beds occupied	95	(89, 100)	94	(86, 100)
Turnover				
% staff turnover last 12 months	14	(8, 21)	9	(4, 17)
% service user turnover last 12 months	82	(55, 113)	92	(56, 120)
Interventions				
Percentage taking clozapine	37	(25, 45)	39	(25, 45)
Percentage taking multiple antipsychotics	0	(0, 0)	0	(0, 0)
Percentage of service users with carer involvement	76	(64, 90)	75	(56, 87)
Percentage of service users who had family intervention in the last year	7	(0, 28)	7	(0, 25)
Percentage who received CBT in the last 12 months	13	(0, 33)	9	(0, 38)
Unit manager “very happy” for their friend/relative to receive care on unit	28	56	27	54
	mean	(SD)	mean	(SD)
QuIRC domain scores (%)				
Living environment	79	(7)	79	(7)
Therapeutic environment	68	(6)	71	(5)
Treatments and interventions	65	(7)	65	(8)
Self-management and autonomy	75	(6)	77	(7)
Human rights	77	(8)	80	(6)
Recovery based practice	72	(7)	74	(7)
Social inclusion	65	(12)	65	(11)

mean used when data Normally distributed, median when skewed

Table 3 shows participant outcomes at 12 month follow-up. Over half (56 %) were successfully discharged and a further 14 % were considered ready for discharge but no suitable vacancy in supported accommodation had been identified for them. There was a small improvement in mean staff ratings of patients’ social functioning (Life Skills Profile) from 128 to 132. Global Assessment of Functioning and Time Use Diary (activity) ratings also improved slightly over the 12 months. Those who were successfully discharged/ready for discharge had a higher mean score on all three ratings at recruitment compared to those who remained in the unit (difference in mean Life Skills Profile 13.78 [95 % CI 11.04, 16.52], Time Use Diary 10.51 [95 % CI 7.50, 13.52], Global Assessment of Functioning 7.29 [95 % CI 5.68, 8.91]).

**Table 3:** Participant outcomes at 12 months (*N* = 329)

	Baseline		12 month follow-up	
	mean	(SD)	n or mean	% or (SD)
Discharged from the rehabilitation unit			219	66
Ready to be discharged from the rehabilitation unit			48	14
Successfully discharged from the rehabilitation unit (*n* = 339)			187	56
Successfully discharged or ready to be discharged from the rehabilitation unit			235	71
Length of admission (months) median (IQR)	18	(9, 38)	24	(15, 48)
Length of admission in the rehabilitation unit (months) median (IQR)	7	(3, 15)	16	(10, 23)
Life Skills Profile score	128	(15)	132	(15)
Global Assessment of Functioning score	53	(8)	56	(9)
Time Use Diary (activity) score	49	(11)	51	(15)

mean used when data Normally distributed, median when skewed

Table 4 shows the results of a) the regression analysis investigating the association between the quality of the unit as assessed using the QuIRC and patients’ social function (Life Skills Profile score) at 12 month follow-up. None of the QuIRC domain scores appeared to be associated with social function. b) the regression analysis investigating the association between unit quality and length of admission in the rehabilitation unit. The small size of the coefficients and the confidence limits suggest that none of the QuIRC domains had clinically important associations with length of admission in the rehabilitation unit.

**Table 4:** Association between unit quality (QuIRC domain scores) and patients social function (Life Skills Profile score) and length of admission in the rehabilitation unit at 12 month follow-up

	Adjusted^a^		Unadjusted	
QuIRC domain	Coefficient or odds ratio	95 % confidence interval	Coefficient or odds ratio	95 % confidence interval
Life skills profile				
Living environment	−0.02	(-0.31, 0.27)	0.11	(-0.16, 0.38)
Therapeutic environment	−0.06	(-0.41, 0.28)	−0.11	(-0.43, 0.21)
Treatments and interventions	−0.18	(-0.45, 0.08)	−0.20	(-0.45, 0.04)
Self-management and autonomy	−0.03	(-0.34, 0.27)	0.10	(-0.19, 0.38)
Human rights	−0.05	(-0.30, 0.21)	0.03	(-0.21, 0.27)
Recovery based practice	−0.09	(-0.38, 0.20)	−0.04	(-0.32, 0.24)
Social inclusion	−0.06	(-0.23, 0.11)	−0.06	(-0.22, 0.09)
Log_e_ length of admission (rehab unit)				
Living environment	0.001	(-0.006, 0.009)	0.005	(-0.014, 0.024)
Therapeutic environment	0.004	(-0.004, 0.013)	−0.010	(-0.032, 0.013)
Treatments and interventions	0.001	(-0.006, 0.008)	−0.003	(-0.021, 0.014)
Self-management and autonomy	0.003	(-0.004, 0.010)	−0.001	(-0.021, 0.019)
Human rights	0.005	(-0.002, 0.011)	0.001	(-0.016, 0.018)
Recovery based practice	−0.000	(-0.007, 0.007)	−0.014	(-0.033, 0.005)
Social inclusion	0.001	(-0.003, 0.005)	−0.003	(-0.014, 0.008)

^a^Adjusted for: age; sex; length of illness; Mental Illness Needs Index (MINI) score; baseline measure of the outcome, risk history (assault on others in the past two years), percentage of service users on the unit detained (unit level variable) p Special Problems Rating Scale (SPRS) score; Clinician Alcohol and Drug Scale (CADS) score

The results of the further multivariable exploratory analyses are shown in Tables 5 and 6. Table 5 shows the variables associated with successful discharge at 12 month follow-up. The communication sub-scale of the Life Skills Profile (which assessed service users’ social skills), the Time Use Diary score (which assessed service users’ level of activity) and the Recovery Based Practice domain of the QuIRC (which assesses the unit’s performance on this aspect of care) were found to be positively associated with successful discharge. The length of patients’ current admission and the percentage of patients in the unit who had had CBT in the 12 months prior to recruitment were associated with a reduced chance of successful discharge.

**Table 5:** Multivariable analysis of predictors of successful discharge

	Odds ratio	95 % confidence interval
Length of current admission (months)	0.99	(0.99, 1.00)
Life Skills Profile communication subscale score	1.13	(1.04, 1.24)
Time Use Diary (activity) score	1.03	(1.01, 1.05)
QuIRC Recovery Based Practice domain score (%)	1.04	(1.00, 1.08)
% service users in the unit who received CBT in the year before recruitment	0.99	(0.98, 1.00)

**Table 6:** Multivariable analysis of predictors of successful discharge and/or readiness for discharge

	Odds ratio	95 % confidence interval
Any history of fire setting	0.35	(0.13, 0.92)
Any self-harm	2.02	(1.16, 3.51)
Length of current admission	0.99	(0.99, 1.00)
Time Use Diary (activity) score	1.05	(1.02, 1.07)

When participants who were ready for discharge and awaiting a suitable placement in the community were included in the analysis, along with those who had achieved a successful discharge at 12 month follow-up, length of current admission was, again, found to be associated with a reduced chance of successful discharge/readiness for discharge, as was any history of fire setting. Patients’ level of activity at recruitment (Time Use Diary score) and a history of self-harm were positively associated with successful discharge/readiness for discharge (Table 6).

All participants used some services at baseline and all but three at follow-up. Service use generally declined over the 12 month follow-up period with the exception of the percentage of service users having contacts with support workers, which stayed around the same, and the percentage having contact with care co-ordinators which underwent a large increase (Table 7). Of those having contact with nurses, the number of hours decreased substantially, while for support workers the opposite was evident. There was a statistically significant reduction in the service costs over the time horizon of the study of £710 (95 % CI £514 to £888). This decrease was largely due to a reduction in nurse costs.

**Table 7:** Use and cost of services at baseline and 12 month follow-up

	Baseline			Follow-up		
Service	% using service	Mean (SE) contacts^b^	Mean (SE) cost (£s)	% using service	Mean (SE) contacts^b^	Mean (SE) cost (£s)
Psychiatrist	97	2.1 (0.1)	245 (9)	66	1.7 (0.1)	134 (9)
Other medical specialist	44	2.9 (0.1)	135 (10)	25	2.3 (0.2)	61 (8)
Clinical Psychologist	19	3.2 (0.2)	36 (5)	11	2.4 (0.3)	15 (3)
Occupational Therapist	57	7.9 (0.4)	31 (2)	23	6.2 (0.7)	11 (1)
Social Worker	7	1.8 (0.3)	15 (4)	10	1.8 (0.2)	21 (4)
Counsellor/psychotherapist	0	4 (.)	1 (1)	1	7 (1)	2 (2)
Volunteer	8	4.1 (0.6)	2 (0)	2	3.3 (0.4)	0 (0)
Art/music/dance therapist	2	3.8 (0.7)	1 (0)	2	3.5 (0.4)	1 (1)
Care coordinator	45	2.3 (0.1)	27 (2)	62	2.8 (0.2)	44 (4)
Advocate	2	2.1 (0.5)	1 (0)	1	1.2 (0.4)	4 (1)
Probation officer	1	2.5 (1.5)	2 (1)	0	4 (.)	1 (1)
Other	12	3.5 (0.5)	24 (7)	6	4.7 (1.2)	14 (6)
Nurse^a^	99	16.4 (0.6)	1216 (46)	90	9.2 (0.6)	678 (47)
Support worker^a^	64	11.8 (0.9)	204 (13)	63	22.7(2.7)	241 (16)
Total cost			1938 (56)			1229 (66)

^a^Number of hours

^b^among those using the service

The regression results suggested that, other things equal, male service users and white service users had a lower amount of service use at baseline (Table 8). Better functioning, as assessed by Global Assessment of Functioning score at baseline, was negatively associated with both baseline and follow-up costs. Service users with more challenging behaviours on the Type D sub-scale of the Special Problems Rating Scale (self-harm and suicidal risk), reported a higher number of costly contacts at baseline but the effect on follow-up costs was not statistically significant. There was a trend towards higher follow-up costs for those patients with Type A sub-scale behaviours (violence to others including arson) at baseline.

**Table 8:** Impact of baseline service user characteristics on costs at baseline and follow-up

	Baseline costs (*N* = 351)			Follow-up costs (*N* = 326)		
Variable	Coefficient	SE	95 % CI	Coefficient	SE	95 % CI
Male	−280.3	126.7	(-528.6, -32)	−206.8	166.4	(-532.9, 119.3)
White	−423.4	209.7	(-834.3, -12.4)	312.9	276.2	(-228.4, 854.3)
GAF score	−15.7	8	(-31.4, -0.1)	−31.1	13.5	(-57.6, -4.6)
Age	2.4	4.8	(-7.1, 11.9)	2.6	7.4	(-11.9, 17.1)
Type D behaviours (SPRS)	188.4	91.7	(8.7, 368.2)	133.7	122.7	(-106.8, 374.3)
Type A behaviours (SPRS)	154.9	122.5	(-85.2, 395.1)	329.9	168.6	(-0.6, 660.4)
Involuntarily admitted	38.5	134.8	(-225.7, 302.6)	40.5	165.1	(-283.1, 364.1)
Baseline cost				0	0.1	(-0.1, 0.2)
Constant	3156.6	608.8	(1963.3, 4349.9)	2607.5	914.1	(815.8, 4399.2)

*SPRS* Special Problems Rating Scale (a measure of challenging behaviours). Type A sub-scale assesses risk of violence and arson. Type D sub-scale includes assessment of self-harm and suicide risk

## Discussion

This cohort study investigated outcomes and costs for users of inpatient mental health rehabilitation units that were assessed as being above median quality during our previous national survey [9]. By including better performing units we aimed to identify the aspects of good quality care that were most likely to be associated with supporting patients to improve in their social function and achieve successful community discharge, the main aim of rehabilitation services [24].

### Main findings

Over half the patients achieved a successful community discharge within the 12 month follow-up period and there were small improvements in the ratings of social function. The multivariable regression models did not appear to identify any associations between the seven QuIRC domains assessing the quality of care provided in the units and better clinical outcomes for patients (social function or length of admission in the rehabiliation unit). However, a number of potential factors that were associated with successful discharge were identified, (albeit with relatively small odds ratios). This analysis was repeated including patients who were considered by staff to be ready for discharge but who were awaiting a suitable community placement, in order to avoid bias due to lack of availability of appropriate local supported accommodation. Both analyses found that the degree to which patients were engaged in activities at recruitment was positively associated with successful discharge/readiness for discharge but those who had been in hospital longer were less likely to achieve this positive outcome. In the first model, patients’ social skills at recruitment (the communication sub-scale of the Life Skills Profile) were also found to be positively associated with successful discharge, as was the degree to which the rehabiliation unit operated with a “recovery” orientation.

Although the negative association between fire setting and discharge/readiness for discharge is not surprising, less easy to explain is the negative association between receiving CBT and successful discharge. These findings are discussed further below.

The cost analysis showed that there was a decrease in costs of care over the 12 months of the cohort study, though the cost of contacts with support workers remained more or less stable and the costs of contacts with care co-ordinators increased. The majority of staff in supported accommodation that most service users moved on to were support workers, explaining the stability of costs associated with this staff group. Quality of care was not associated with costs of care when adjusted for service user age, gender and social functioning. Less severe symptoms and higher functioning (higher scores on the Global Assessment of Functioning) were associated with lower costs of care, presumably because service users who were less unwell had less need of staff support. This is something that has been demonstrated previously [25]. Service users with a history of fire setting were less likely to be discharged in our sample. Therefore, there was a trend towards higher costs at follow-up among those with problems on the Special Problems Rating Subscale that included this behaviour.

### Clincal implications

Our results are important in helping to inform the practice and interventions that are most likely to help people with complex mental health needs progress in their rehabilitation. The finding that patients’ activity at recruitment into the study was associated with successful discharge/readiness for discharge at 12 months supports our decision to develop and test a staff training intervention aimed at promoting patient activity during other phases of the REAL study [26]. The association with social skills and successful discharge is also of interest. The evidence for the effectiveness of social skills training for people with schizophrenia has not been considered adequate for NICE [27] to recommend routinely offering it to people with schizophrenia. However, a meta-analysis of 22 trials of social skills training [28] found it to be associated with improvements in psychosocial functioning and negative symptoms, though problems with heterogeneity of methods and reporting of results limited the robustness of the findings [29]. Nevertheless, the Scottish Intercollegiate Guidelines Network guidance on the management of schizophrenia [30] states “social skills training may be considered for individuals diagnosed with schizophrenia who have persisting problems related to social skills.” Further studies are required to investigate the potential benefit of specific social skills training for people with complex mental health problems who are referred to rehabilitation services.

Our finding of a positive association between successful discharge and recovery orientated practice is of particular interest. Recovery orientated practice in mental health services is strongly encouraged by policy makers [31]. It incorporates a focus on therapeutic optimism and collaborative working with patients to agree together the goals of treatment and support, rather than the more traditional approach of a professional led treatment plan with the patient as passive recipient [32, 33]. Mental health rehabilitation services were early adopters of the recovery approach [34] and current commissioning guidance describes them as operating with this style and values [24]. One specific aspect of recovery orientated practice, namely the employment of ex-patients as members of the inpatient staff team occurred in 38 % of units (and one third of units across England in our national survey). The Recovery Based Practice domain of the QuIRC also includes many other aspects of care, including assessment of the degree to which collaborative care planning practices are employed and the therapeutic optimisim of the staff. We believe that our results may provide the first empirical evidence of the possible benefits of recovery orientated practice for people with complex psychosis.

We also identified factors associated with less chance of successful discharge/readiness for discharge. The greater the percentage of patients in the unit who had received CBT in the year before recruitment into the study, the less likely successful discharge was. Whilst this could be interpreted as suggesting a negative effect of CBT, there is strong evidence of its effectiveness in people with psychosis and it is recommended for treatment of this group [27]. A more likely explanation is that patients with the most complex needs, who are hardest to treat, are more likely to receive CBT as part of the range of interventions aimed at improving symptoms and functioning. This explanation concurs with the finding that patients who had been in hospital longer were less likely to achieve successful discharge/readiness for discharge. In other words, those with the most complex and treatment resistant symptoms tend to remain in hospital longer and are, perhaps, more likely to be offered more interventions over time. A possible alternative explanation is that patients who engaged with CBT developed greater insight into their mental health problems but required longer inpatient treatment as a consequence. However, we did not assess insight in this study and must stress that this possible association is purely hypothetical.

A history of fire setting was also associated with less chance of successful discharge/readiness for discharge and greater costs of care, although only 7 % of the cohort had such a history. Challenging and dangerous behaviours have previously been noted to make individuals difficult to discharge from hospital [5, 8]. Arson is an especially challenging behaviour and many supported accommodation providers are, understandably, reluctant to offer placements to people with this kind of serious risk history. Conversely, we found that a history of self-harm (which had occurred for 41 % of the cohort) was associated with a greater chance of successful discharge/readiness for discharge. This seems a rather paradoxical finding. Perhaps those who self-harm have less severe negative symptoms and are more motivated to act (albeit in a detrimental manner) than those with more severe negative symptoms whose level of function is so poor that it impedes community discharge. Self-harm may also indicate the presence of mood symptoms [35] which are generally associated with a better prognosis than negative symptoms alone. It should also be borne in mind that this factor included self-harm at any point in the person’s history, and these acts may have been many years earlier.

The fact that 14 % (1 in 7) of patients whom staff considered ready for discharge could not leave the unit because no suitable community accommodation was available is concerning. This represents an inefficient use of resources and needs to be addressed urgently to ensure that patients are supported in the least restrictive environment appropriate to their needs. More investment in community based supported accommodation is therefore required. This should include specialist accommodation for the small percentage of service users whose challenging behaviours such as a history of fire setting, impede move-on.

### Strengths and limitations

Our study was only able to report associations between service and service user characteristics and outcomes. Since mental health rehabiliation services have been in place across England for many years, randomisation was not possible and there are no suitable comparison services that could be used for a case control design. The only feasible approach to evaluation therefore, was an observational study. Whilst our analyses were exploratory, we can have some confidence in our findings. We recruited a large sample from across most of the better performing inpatient mental health rehabilitation units in England. Our follow-up rate was excellent, with primary outcome data on successful discharge collected on over 90 % of our cohort. This is also a “hard” dichotomous outcome which does not rely on subjective opinion. Our decision to use staff rated outcomes also minimised the amount of missing data on patients’ social functioning at 12 month follow-up. However, inclusion of patient rated outcomes, such as quality of life and satisfaction with treatment and support, would have allowed us to report on a more comprehensive range of perspectives on the concept of “meaningful” clinical outcome.

Nevertheless, our results appear to support the therapeutic optimism that is enouraged in mental health rehabilitation services. The majority of patients in our study were successfully discharged to the community within our 12 month follow-up period (without readmission or community placement breakdown), despite the severity and complexity of their mental health problems that had led to their referral to these specialist services. Mental health rehabilitation services are therefore succeeding with an especially complex group and reducing the costs of care through their input. We found that successful discharge was associated with units operating an approach that incorporated a recovery orientation. Higher levels of patient activity and social skills were also associated with a greater chance of successful discharge. Patients with more complex needs and challenging behaviours (specifically, fire setting) were less likely to achieve successful discharge. Our findings suggest that further research is needed to identify effective interventions that enhance recovery orientated practice, patient activities and social skills in these settings.

Whilst our study was carried out in better performing services in England, the results have obvious relevance for lower quality units, not just in the UK, but in other countries where the quality of care may differ.

## Conclusions

Higher quality inpatient mental health rehabilitation services in England are able to successfully discharge over half their patients within around 18 months, with associated reductions in the costs of care. Provision of recovery orientated practice that promotes patients’ social skills and activities may be important in improving the effectiveness of services for people with complex psychosis.

### Ethics approval and consent to participate

The REAL study was approved by the South East Essex Research Ethics Committee (Ref. 09/H1102/45). All participants provided written informed consent to take part including their willingness for anonymised findings to be used in publications and reports.

### Consent for publication

All authors approved the final version of the manuscript and agreed their accountability in ensuring that any questions related to the accuracy or integrity of any part of the work are appropriately investigated and resolved.

### Availability of data and materials

All data supporting our findings will be shared on request.

## Abbreviations

QuIRC: Quality Indicator for Rehabilitative Care
REAL: Rehabilitation Effectiveness for Activities for Life
UK: United Kingdom
